# Experiences with online group work among master’s students in health sciences: a qualitative study

**DOI:** 10.1186/s12909-025-07845-w

**Published:** 2025-09-25

**Authors:** Live Edvardsen Tonheim, Marianne Molin, Asgeir Brevik, Lisa Garnweidner-Holme

**Affiliations:** 1 Department of Nursing and Health Promotion, Faculty of Health Science, Oslo Metropolitan University, Oslo, 0130 Norway; 2 Department of Health and Exercise, School of Health Sciences, Kristiania University of Applied Sciences, Oslo, 0153 Norway

**Keywords:** Online group work, Health science education, Online education, Higher education, Student group work, Students’ experiences

## Abstract

**Background:**

Group work is commonly used in health science education to foster active learning and developing teamwork skills. Following the online transition in higher education, group work is increasingly conducted online. Although, group work offers online students a valuable arena to interact with and learn from fellow students, research indicates challenges for successful online collaboration. Few studies have focused on facilitators and barriers to online group work as their primary objective, and even fewer within health sciences providing in-depth knowledge of the students’ perspective. Therefore, we aimed to explore health science master’s students’ experiences of facilitators and barriers to online group work.

**Methods:**

We conducted individual interviews with master’s students in health sciences enrolled at a Norwegian university. The interviews were conducted from May 13th, 2022, through September 5th, 2022, by videoconference. A semi-structured interview guide was used, and the interviews were analysed guided by Reflexive Thematic Analysis by Braun and Clark.

**Results:**

Fourteen students from eight different health science master’s programmes were interviewed. From the analysis we developed three main themes: “Participating in online group work - a matter of priority”, “Students’ need for continuity in the group” and “Finding the flow in online group work”. These were further described in eight subthemes: “The duality of flexibility” and “The need for valid reasons to prioritise participation”, “The need to get to know the others”, “The need to feel safe in the group”, “Shared responsibility in participation”, “Mastering communication”, “Technical infrastructure and competences”, and “Clear assignments and guidance”.

**Conclusions:**

Based on our findings we believe online group work needs to be adapted to the specific demands of the online setting and have several proposals. One may consider making elements of the group work mandatory to provide continuity in the group. Allocating time for groups to get to know each other and introducing basic guidelines for online group work may foster good group dynamics. Also using assignments that promote shared effort, clearly outlining how the students may benefit from participating, may promote group processes that enhance learning and satisfaction. Hopefully this may encourage students to prioritise participation in online group work.

**Supplementary Information:**

The online version contains supplementary material available at 10.1186/s12909-025-07845-w.

## Background

Group work is a common pedagogical strategy in health science education, used to promote active learning. When working in groups with clinical cases or complex problems, students are encouraged to apply knowledge to practice and learn from each other’s perspectives [1]. Working in student groups has been found to promote deeper understanding [2, 3], critical thinking [2, 4] and academic knowledge [5]. In addition, it may help the students develop communication and teamwork skills essential to health professional work [1].

As online courses have become part of health science education, group work is also increasingly conducted online [6], either during lectures, as part of voluntary or mandatory work assignments, or in exams. The students may communicate and conduct their group work synchronous (in real time), asynchronous (flexible, at any time) [7], or bichronous (a blending of the two) [8], using various online channels such as videoconferences, shared documents, and chats. We included all the modalities above in our understanding of online group work.

Among the benefits of online group work, is the opportunity offered to the students to interact with peers and be part of a learning community [9]. In online courses, such arenas are important to help students connect and form relationships with other students [10]. Connecting with fellow students may in turn contribute to motivation and endurance [10–13] and prevent feelings of isolation and drop-out [10, 12], which are all known challenges to online education. In addition, research have shown that students participate more actively in smaller groups than in larger lectures online [14], and that they value the opportunity to engage in discussions and gain different viewpoints from others [2]. However, implementing group work in online courses, is not without challenges [15]. Some are well-documented from studies on in-person group work such as unequal participation and free-riding, the perception that group work is not worth the time [16], and challenges related to group dynamics and group relationships [3]. Other challenges, more specific to online contexts, relate to technical issues with equipment or competence, scheduling and timing issues, and choosing appropriate platforms and tools for collaboration [15].

Although a growing body of literature has addressed online group work in health science education [17, 18], these studies focus on group work within specific learning designs or course evaluations [19]. Few address facilitators and barriers for online group work in master’s courses as their primary objective [19]. Consequently, there is limited in-depth knowledge of health science master’s students’ experiences with online group work. Given the increasing demand for health personnel, it is important to improve online master’s programmes that enable professionals to pursue further qualifications while working, making such research essential. To the best of our knowledge, this is the first study to use individual interviews to explore health science master’s students’ perspectives on facilitators and barriers to successful online group work. Our study aimed to answer the following research question: What are health science master’s students’ experiences with facilitators and barriers to online group work?

## Methods

We conducted a qualitative interview study, and used the Consolidated Criteria for Reporting Qualitative Studies (COREQ) [20] as a guideline to promote transparency and ensure that important aspects of the process were reported.

### Study setting

The study was conducted among master students in health sciences at a Norwegian university. The students were enrolled during or in the wake of the COVID-19 pandemic in a master’s program that consists of both mandatory, core and specialised, and elective courses.

### Participants

As we aimed to recruit students who had participated in online group work, we used a purposive sampling approach. Students who had attended one of the master’s programme’s mandatory core courses that were offered solely online were invited to participate through Canvas announcements and via student e-mail addresses. In addition, we asked student representatives from each master’s specialisation to post information about the study in closed Facebook groups. Eleven participants were recruited by these efforts. Three more participants were recruited using the snowball sampling method through former participants. All students were offered a universal gift card (500 NOK) for their participation. Students who had been previously tutored by the author conducting the interviews were excluded. In total, we recruited 14 participants, aged 25 to 65 years.

### Ethical considerations

The study was conducted in accordance with the ethical research guidelines of the university and was evaluated by the Norwegian Agency for Shared Services in Education and Research (SIKT) (reference number 657113). All participants were given oral and written information about the study and their right to withdraw from the study without any reason. Written consent was obtained prior to the interviews, and the participants gave additional oral consent before the audio recordings began. All data were anonymised.

### Interview guide

The research group developed a semi-structured interview guide with open questions centred on the research question (Appendix 1). The interview guide was developed to enable an inductive exploration of students’ experiences concerning the facilitators and barriers of online group work, and the context of these experiences. The interview guide was piloted by the first author with two students at the health science master’s programme at the beginning of May 2022. All authors discussed and decided on adjustments based on the transcriptions and the interviewer’s account of the pilot interviews.

### Interviews

All interviews were conducted from May 13th, 2022, through September 5th, 2022, by the first author. The interviews were conducted using Zoom and lasted between 30 min and 1 h. Video was turned on in all interviews, but only audio was recorded using “Nettskjema diktafon app”, a survey and dictaphone solution developed and hosted by the University of Oslo (nettskjema@usit.uio.no). The app provides access to recordings only via a secure webpage. All recordings were deleted after the transcription.

### Analysis

We analysed the data guided by Reflexive Thematic Analysis (RTA) [21, 22]. The analysis was situated in a constructionist epistemology as our aim was to develop themes that in addition to recurrence also considered the meaningfulness and importance expressed by the students. Because we wanted to explore the students’ experiences from their point of view we chose an experiential approach [23]. Furthermore, we wished to explore our data freely to present the meanings as communicated by our participants. Braun and Clarke describe this as a more inductive approach, as we used open and data driven coding as opposed to a pre-existing theoretical construct [21, 23]. However, as further explained by Braun and Clarke [21] and discussed by Byrne [23] RTA is never exclusively inductive, as our preconceptions, assumptions and research questions shaped our decisions and interpretations throughout the analysis.

The same researcher conducted and transcribed all the interviews verbatim. Repeated listening to the recordings increased accuracy in the transcription and allowed the researcher to start to familiarise with the data. This first phase in the RTA was continued through a thorough and repeated reading of the interviews by the first author, noting initial thoughts in memos. Following, the other authors read the transcriptions to increase accuracy by adding their reflexions. The data was coded systematically (second phase) using a predominantly semantic approach, as we wanted to stay close to the explicit surface meaning communicated by students [21]. Following the recommendations by Braun and Clarke [21], the data was coded twice, the second time in reverse order to allow for further clarity and refinement of codes. All coding was performed in NVivo (14) [24]. The first author generated the initial themes (third phase) by collating codes that seemed to be related to the same key concepts. Initial themes were reviewed and discussed in meetings with all the authors, which provided feedback for reflection and further development. Based on these meetings, the potential themes were reviewed and some were discarded, while others were divided or combined to increase clarity and meaningfulness. The fourth phase of developing and reviewing themes also involved re-coding where necessary, moving back and forth between the dataset, codes and themes to ensure that we stayed close to the data while at the same time presenting the key patterns in the entire dataset. Refining, defining and naming in the fifth phase and writing up in the sixth phase was partly blended as the process of writing up also contributed to finer adjustments. Records of the development of themes and codes were kept in NVivo, mind maps, and tables. Throughout the whole process, the authors repeatedly met to discuss developments.

### Reflexivity

All the authors in the research team had previous experience tutoring online groups in health science education, although to varying degrees. While the second, third and fourth authors were experienced educators and researchers that were practiced in developing group assignments and online courses in higher education, the first author had experienced online group work as an online student for several years. Following RTA we kept aware of our different experiences, assumptions and expectations, using our different perspectives in reflexions and discussions to develop a compelling analysis.

## Results

Fourteen students aged 25 to 56 years participated in interviews (Table 1). The students were enrolled in eight different master’s programmes in health sciences, and most worked beside studying. All the students had participated in synchronous group work during online lectures (breakout rooms) and 11 of 14 students had also participated in longer term group work such as work assignments, seminars or project exams which required a combination of synchronous and asynchronous collaboration toward a shared product. While all had experienced online group work in the same mandatory course, they also had experiences with online group work from other courses in the master’s program.

**Table 1:** Relevant background information about the participants

Specialisation – master’s in health sciences		Full time/part time	Age (Median/Range)	Gender (Woman/Man)	Working beside studying (Yes/No)
Empowerment and Health Promotion	2	Full time	31/25–56	13/1	11/3
Occupational Therapy	1	Part time			
Nutrition Competencies for Health Professionals	5	Full time			
Physiotherapy for Children and Adolescents	1	Part time			
Physiotherapy for Musculoskeletal Health	1	Part time			
Cancer Nursing	1	Full time			
Psychomotor Physiotherapy	2	Part time			
Nursing; Clinical Research and Professional Development	1	Part time			

**Fig. 1:**
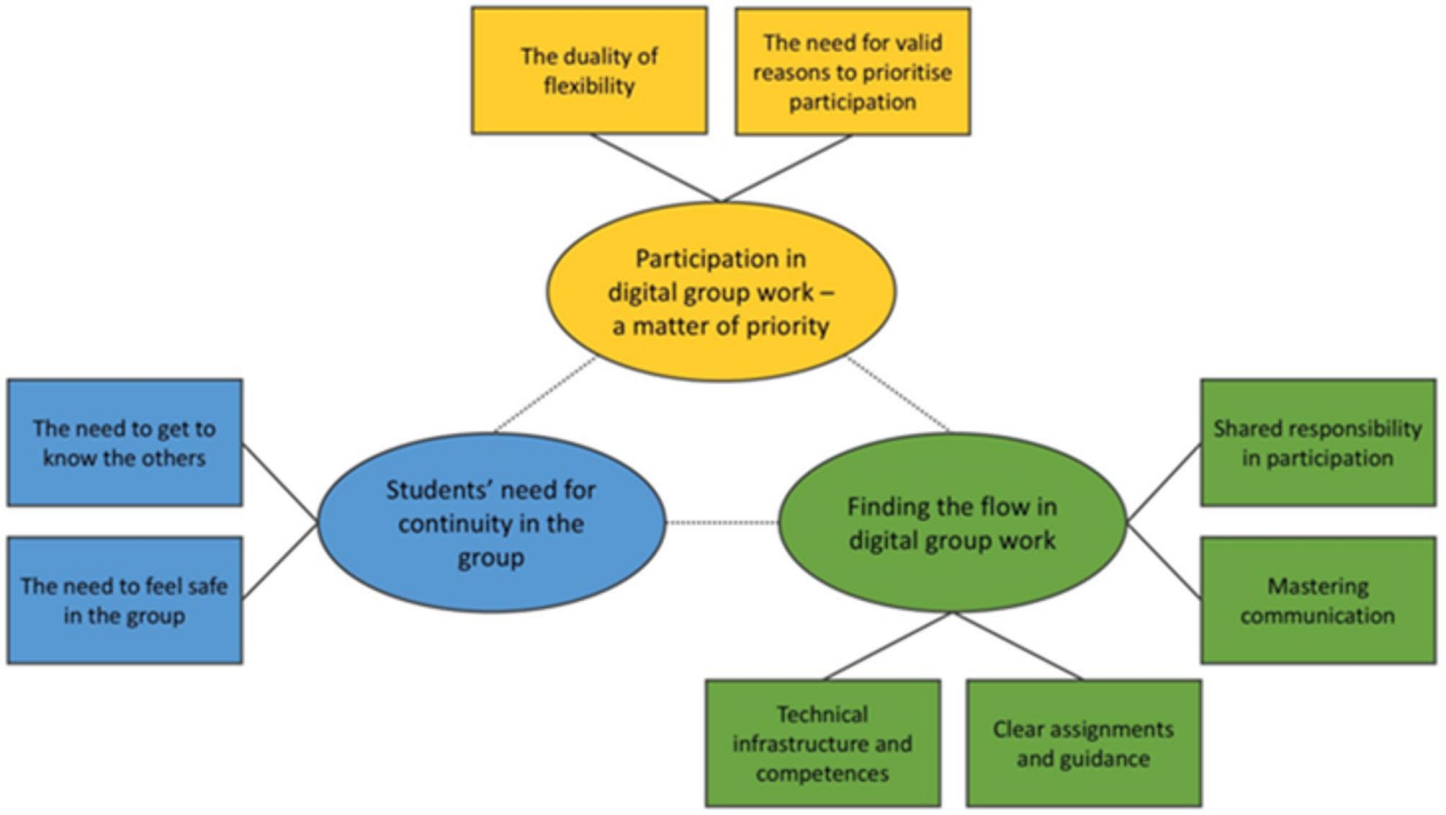
Themes (ovals) and subthemes (squares). Each theme with respective subthemes is represented by a different colour and relationship with the subthemes are indicted by solid lines. The dotted lines indicate that the themes mutually influence one other

We developed three themes and eight subthemes (later referred to in bold text) from the analysis (Fig. 1), which will be presented as follows.

### Participating in online group work - a matter of priority

This theme encompassed two subthemes: the duality of flexibility in online group work and the need for valid reasons to prioritise participation. The students clearly valued the flexibility of online group work.

*“… people do have different lives. Like myself*,* I’m very happy with flexibility because I move around a lot*,* travel a lot*,* so it’s good to kind of just having my school desk with me wherever I go. And then I think*,* for those who have children or families or who are a bit older who can’t move around or don’t have the opportunity to do so*,* it’s a super advantage with the online*,* also with online group work.” (S12)*.

Flexibility was described as convenient and timesaving, and the general impression among the students was that it allowed everyone to meet at any time. Nevertheless, scheduling group meetings was experienced as a major challenge, attributed to the group members’ other commitments in everyday life. This was described by one of the full-time students as follows:

*“…. So*,* then it’s a bit difficult to find a common time when everyone can meet on Zoom…… That’s perhaps kind of the recurring problem*,* finding time that suits everyone” (S14)*.

Similarly, students could end up alone in their group in synchronous lectures if their fellow group members did not attend, and another full-time student shared their experience:

“*… you’re not guaranteed that those you’re put in a group with are present because you don’t… well*,* because they have other things they prioritise…. whether it’s work or whatever*,* I don’t know. But then I ended up changing groups.” (S11)*.

The students’ accounts suggested that due to the flexibility of online education, participation in online group work became a matter of choice and thus a question of priority. One of the students reflected upon whether this could be a consequence of students’ reasons for choosing online studies in the first place:

*“….that’s perhaps a bit the main factor… because those who do online studies may not have ((small laugh)) much time to begin with to be able to be at school physically…” (S14)*.

To prioritise participation in online group work, the students expressed a need for valid reasons. Among these were commitment to the group and motivation. Although, the latter was perceived important, it was not necessarily sufficient, and one of the students expressed their frustration: *“In the beginning*,* I was motivated by the study*,* but I didn’t understand why I should join.” (S05)*.

However, both full-time and part-time students were clear about what they regarded as the most effective incentive:

“*When I think about it now*,* I think the fact that things have been compulsory have perhaps brought out the best results” (S14)*.

*“Yes*,* it’s kind of the fact that it’s an obligation because there’s a work requirement that needs to be approved before you can sit for the exam and then everyone shows up…… and is present.” (S07)*.

### Students’ need for continuity in groups

The students experienced that having fixed groups and meeting regularly facilitated the online group work. One student explained it like this:

*“…. it was probably a bit that you got that continuity in the group. And that*,* I would say in a way made it work*,* even the presentation task*,* and the work requirement also worked well*,* because then it was like the group you had been working with*,* together with over time” (S03)*.

Continuity in the groups enabled the students to meet two central needs: the need to get to know the others and the need to feel safe in the group. According to the students’ experiences, it seemed that establishing relationships required more time in the online environment than in the physical.

*“…in a way it may take some time before you get to know each other through the online world because it’s more challenging than if you had been physically present.” (S11)*.

*“…… eventually you get to know each other online….*,* but you do get to know each other. And that sort of makes the cooperation better.” (S09).*

In addition to facilitating cooperation, getting to know the others was pivotal to developing a sense of commitment to the group, and as one student in their twenties explained:

*“It happens a bit over time*,* that you get to know each other and actually… in my group now I feel that*,* over time we have somehow*,* yes that thing about commitment… I feel it has happened quite naturally” (S05)*.

Getting to know the others and feeling safe was closely connected, and the importance of feeling safe was strongly emphasised. As a parttime student said *“… it is a bit daunting*,* I have to say. It is a bit daunting to have to speak to strangers” (S04).* Another student, in their thirties, explained it thus:

*“… Feeling safe is quite important*,* at least for my part*,* to contribute…so I notice that I am more involved in group tasks or discussions with people that I’ve been in a group with before and have gotten to know a bit.” (S03)*.

### Finding the flow in online group work

The theme of finding the flow could be described as the ‘x-factor’ of well-functioning online group work. In these group works, students felt lucky with their group, and experienced that they ‘worked well together’. Based on the students’ accounts, ‘flow’ in the group work was characterised by what we developed as subthemes: shared responsibility in participation and mastering communication in online group work, processes that were supported by the two other subthemes clear assignments and guidance and technical infrastructure and competence.

Shared responsibility in participation was experienced when *“…everyone contributes*,* often in slightly different areas.” (S05)*. Similarly, “*…everyone has cooperated…“ (S16)* seemed to be a recurring answer to why students were happy with their group. Students also valued that the group worked as a whole. When explaining how this was achieved, a student said *“….and in that way*,* I think we just sort of built*,* built something together…” (S06).*

Although, the students found dividing tasks convenient, and sometimes a starting point for further collaboration it was also seen as the opposite of a collective effort attributed to the online environment, as a student in their thirties recounted:

*“… instead of having a holistic uh: approach to it like*…*when you work*,* non-digitally it is easier to make the task more: “the whole group” (S15)*.

Similar comparisons between online and in-person group work were frequently brought up, and the students experienced that the challenges in passivity also experienced in in-person group work were more prominent in the online. One student expressed it as follows:

*“Sometimes*,* one can get frustrated that even though a person is present*,* they are not truly present. This can also happen physically. However*,* I think it’s easier to sneak away online than physically” (S11)*.

The students described how one can be anonymous in online environments, and how that affected participation, as shown by the following reflections of a student in their twenties:

*“Yes*,* it could be because if one’s screen is black*,* no one sees you*,* and you can just sit there like a shadow… It’s a bit easier to be anonymous and*,* yeah*,* just sit and watch and not participate. It’s easier to not participate” (S10)*.

Finding the flow in the online group work was also dependent upon mastering communication. When organising the group work students described that in well-functioning groups *“… we are good at sending messages and planning” (S16).* Key to good communication in organising the group was establishing and maintaining contact. The students stressed that the main challenge was to get everyone to answer but found social media an efficient channel to get in touch and keep each other informed *“…either over FaceTime as a group or on Messenger where we have just created our own*,* yes*,* online forums just so you can meet up and get the work done there” (S13)*.

Finding the flow in online synchronous group meetings was emphasised as particularly challenging. The students thought it *“… a bit unnatural….to talk in that way. At least if everyone has their microphones off and has to turn it on before they start talking and turn it off and on for the next person*,* so it’s a bit unnatural*,* it is….” (S10)*.

Especially those who were not among the most talkative or needed to ponder before sharing their opinion found the turn-taking dynamics challenging. As a student in their forties described it:

*“…when we talk*,* everyone has to be quiet when someone is talking… And if one person is very talkative ((small laugh)) it’s like*,* you don’t get a word in*,* so it’s things like that*,* but like in in-person there is much more flow*,* of course*,* and eye contact is important. You don’t have that in online teaching*,* online group work. You don’t have that*.” (*S09)*.

However, the students meant that this challenge could be somewhat mitigated by turning on the camera because when: *“….we have the camera on… I feel that for my own part*,* it contributes to you engaging a bit more*,* that we see each other and that we can communicate non-verbally as well” (S07)*.

Group size was also found to influence the flow in the group. Although the students found larger groups to be less vulnerable to drop-out and low attendance, most preferred a smaller group.

“*…The larger the group*,* the harder it is to coordinate….…So*,* I find that the smaller the groups*,* the easier it is to participate*,* in both group discussions and to voice your opinion*,* yes. It’s easier to be heard and hear others when the group is smaller… I think three works*,* five I think is too many” (S15)*.

The students expressed the necessity of clear assignments and guidance from educators to support both participation and communication. They particularly wanted specific tasks or questions as opposed to open discussion topics. Without a clear assignment, the discussion would halt or steer off course, as described by one of the part-time students:

*“It can easily just become a bit of a muddled discussion if the question isn’t very clear from the lecturer or if one doesn’t understand what the question is about*,* then it can just end up being about other things…not because you*,* don’t want to talk about the academic stuff but simply because you didn’t understand it.” (S13)*.

Especially in synchronous group discussions, the students depended on quickly understanding the task and emphasised that “*…having teachers*,* lecturers*,* and advisers who can come in and help us and clarify a bit*,* is also super important.” (S04)*. Furthermore, they agreed that rapid feedback was key to keeping the discussion going and *“to maintain this flow again*,* that it doesn’t take a long time before this teacher comes around when we have asked for help…” (S10)*.

In addition to be available for questions, students also wanted guidance on how to work in groups and *“… wished that a bit more time was spent initially by the lecturer explaining how to work in groups….” (S06).* Although, thought of as elementary, the students acknowledged the need *“…. to actually get these somewhat banal communication rules and*,* strategies for how one wants group work to be.” (S13)*.

Technical infrastructures and competences were recurringly highlighted as prerequisites to maintain participation and communication in the group. In addition to having a good camera and microphone, high speed internet connection was crucial to the flow in communication. One of the students explained that this was important because:

*“… if you have poor internet then it’s kind of not your fault*,* but of course*,* it does something with the group dynamics if you are constantly freezing or you drop out……If you’re in a good discussion and then suddenly the whole thing stops and it’s like «Did anyone catch that». It’s very*,* frustrating.” (S15)*.

Also, students’ accounts suggested that not all felt they had sufficient competence to use their technical equipment, or to fully exploit the possibilities of the online learning resources used (e.g. Zoom, shared documents). Especially students who had returned to study after years of working as health professionals felt they could not meet the expectations of digital competence. However, this was solved if someone in the group knew ‘how to’ and could help the others, as expressed by one of the students in their forties:

*“There were so many possibilities that I wasn’t*,* familiar with*,* or knew about ((little laughter)) …. which were untapped*,* would have been untapped resources had it not been for someone in the group knowing” (S06)*.

## Discussion

While each student in our study had their own unique experience with online group work, our analysis revealed multiple factors acting together as facilitators and barriers. The themes we developed are the central conditions that influenced the students’ experiences of online group work.

### Participating in online group work – a matter of priority

Among the most frequently mentioned advantages of online education, also emphasised by the students in our study, is the flexibility to study wherever and whenever [25–27]. Flexibility allows students to accommodate their studies around other commitments such as paid jobs or family life [10] which aligns with national [28] and global goals to make higher education available for all regardless of life-conditions [29, 30]. While this may benefit the individual student, our study shows that it can be a barrier to online group work when accommodating the group work to fit the commitments of all group members. In Norway approximately 68% of full-time students combine their studies with paid jobs, which corresponds to 11 of 14 of our study participants working beside studying. Similar tendencies are seen in the UK and throughout Europe, where 56% [31] and 59% [32] of students, respectively, are employed in regular jobs. Because time spent working inevitably reduces time available for studying [32], conflicting priorities may arise, an experience shared by the students we interviewed. They commonly felt that studying, especially group work, was a low priority for some, leading to absences from synchronous group work in online lectures and scheduling challenges in larger group assignments. This left committed members frustrated, and sometimes working alone. Consistent with our findings, previous research have recognised scheduling challenges as a main barrier to online group work, both within health sciences [19] and across disciplines [15]. Mathisen and Søreng [33] noted that time constraints, due to work and family commitments, was a primary reason for students to choose independent work over group work. Likewise, Brindley et al. [14] argued that online students seeking flexibility may see group work as an impediment to their progress and balk at participating. Prioritising between studies and work may be specially challenging for students in similar settings to ours. Health science master’s students in Norway typically return to complete their master’s degree after years of clinical practice. As more adult learners than bachelor’s students, they often have family commitments and rely on continuing to work either full or part time alongside their studies. In addition, as health professionals they have the added challenge of irregular working hours or shift work. For this reason, health science master’s students are likely to continue combining online studies and other commitments, and thus the question becomes what is required for these students to choose to invest time in online group work and prioritise participation? According to our results, the students need valid reasons to choose to participate. While they agreed with previous research, identifying individual motivation to learn or do well as a potential facilitator, this was not enough if the students did not understand why they should participate. Acknowledging that online students need to understand how they can benefit from participating in group work, Brindley et al. [14] propose making group tasks that are relevant to the students and making the relevance of the group work clear. The latter was the main objective of a study by Kelly et al. [34], which found a positive effect of a utility-value intervention aiming to make students aware of the advantages of group work for academic success and future career. On a similar note, Hill et al. stated that “with too much freedom can come too little action” [35]. The students in our study agreed, suggesting compulsory assignments or attendance as key to boost participation. However, one should be aware of the trade-off in increasing obligation, as it would compromise the highly valued flexibility. Loss of flexibility would probably make studying less practicable for adult students with commitments as discussed above, and compulsory attendance could potentially prevent health professionals to pursue a master’s degree in the first place. One possibility could be to use more compulsory group assignments, while keeping attendance voluntary. However, compulsory work may also add to the recognised scheduling challenges, unless groups were assembled according to available study time (evenings, daytime, weekends), or the work organised completely asynchronously.

### Students’ need for continuity in groups

The second main theme addressed the students’ need for continuity in groups. Supported by previous studies within health science education, being in the same group over time may contribute to well-functioning online group work [35–38]. We found that continuity was a prerequisite of “getting to know the others” and “feeling safe to participate” which are important for learning in traditional [39] as well as online group work [19]. However, as our participants described, getting to know the others and building trust takes more time online than in-person [40]. Some of the students in our study attributed this to the lack of informal talk usually occurring in shorter breaks in in-person group work describing how the group would turn off their camera and microphone during breaks. In contrast, students also explained how meeting and communicating regularly with the group led to better group processes and sense of cohesion and commitment. This is in accordance with studies within health science education recommending fixed groups [35–37], arguing that establishing sense of community [35], increasing cohesion [37] and creating stability [36] promote learning [35, 36] and positive group experiences [37]. The value of creating sense of community and cohesion in online collaborative learning has been demonstrated in more than two decades of research employing the Community of Inquiry Framework [9, 41]. The framework’s three fundamental elements include “social presence,” which is characterised by group cohesion and open communication in a trusting environment [9]. While the students we interviewed also mentioned benefits of random groups, such as meeting new people and gaining new perspectives, they said that they were more likely to share their thoughts and opinions in a fixed group. Furthermore, they experienced that most of the allotted time in each new group would be used for introduction because it felt unnatural to jump straight to answering case questions without basic knowledge of the other group members. In fixed groups, however, the students described getting into the task straight away, which was experienced as a much more satisfying and an efficient use of time. Campbell et al. [42] found that while students preferred fixed groups, no difference in learning outcomes was observed between fixed and random groups. In master’s programmes like the one in this study, fixed groups across the entire degree are impractical due to varying specialisations and elective courses. However, fixed groups within individual courses are common as reflected in the research previously discussed [19, 35, 36]. In online lectures, fixed groups can also be practical due to pre-arranged breakout room options in most videoconferencing tools. Still, they may pose a drawback in sessions with variable attendance, as some students may end up alone in their group, as observed in our study. Moreover, it is questionable whether health science students’ preference for fixed groups should be accommodated at all times at the master’s level, as healthcare work often requires quickly establishing communication and trust with new patients and interprofessional teams, making it an essential skill and learning outcome for health science master’s students.

### Finding the flow in online group work

The structures and conditions that lead to good group dynamics are often difficult to pinpoint. Kwon et al. [43] propose that students may attribute early successful group regulation to ‘good chemistry’ without understanding how and why this is achieved. Similarly, our study found that students with positive online group work experiences attributed it to ‘being lucky with the group’, struggling to identify specific reasons. However, when prompted to provide examples, they described group work characterised by active participation from all members and shared responsibility in both task completion and communication. These characteristics also constituted what the students experienced as ‘flow’ in the group work. In these groups, rather than just dividing tasks for individual completion, group members revised and contributed to the others’ work asynchronously and made sure everyone was included in synchronous discussions. Students who believed they had not achieved the level of collaboration they desired, partially attributed this to the online setting, noting that joint processes were perceived as easier to achieve in in-person group work. They explained that online meetings and asynchronous communication enabled anonymity. Donelan and Kear [15] refer to this as “lurking”, suggesting it is a possible explanation for the difficulties in achieving accountability within online groups. However, according to our participants, anonymity could be reduced by establishing and maintaining communication such as through chat groups (e.g., Messenger), enabling camera, and engaging in synchronous meetings. Aligning with Hovlid et al. [36] enabling camera was also highlighted by the students as a key factor in synchronous discussions, helping to alleviate the absence of body language, a common challenge in online group work [18, 36, 44]. Our participants found lack of non-verbal cues made it difficult to determine when to speak, further disrupting the already halted flow caused by the need to mute and unmute the microphone to avoid background noise. To alleviate difficulties with lacking communication and challenges [15, 19, 35, 45], and challenges in establishing and agreeing on a communication channels [46, 47] frequently observed in our study, the students expressed a need for more educator involvement. In line with previous research [14, 19, 48], they explained that ‘finding the flow’ in communication and organisation of the online group work was greatly influenced by the clarity of the assignment and the educators’ guidance. In addition to understanding the assignment, the students emphasised the importance of understanding how to work in online groups, and voiced that they missed guidance from the educators on how to work in a group, as not all individuals possess this knowledge. While this is in line with studies on health science students on undergrad or bachelor’s levels [34, 49],, our findings suggest that it may also be true for master’s students, and that educators should also give instructions on ‘how’ in addition to ‘what’ in group assignments. The students in our study also advised that educators should not take for granted that all students have sufficient digital competence. Although one might expect most are familiar with digital tools like shared documents, Teams, and Zoom, we found this was not always true for health science master’s students returning to university after years in clinical practice. The ability to work in teams is essential in health professional work and collaboration is increasingly taking place through digital health care service systems and digital platforms for clinical collaboration [50]. Thus developing digital collaboration skills is an important learning outcome of group work in health education [1]. Consistent with previous studies [19] the students we interviewed preferred smaller groups of 3–4, explaining that ‘flow’ in work processes and synchronous discussions were easier in smaller groups. However, as observed by Donelan and Kear [15] smaller groups were more vulnerable to drop-out, sometimes resulting in only one or two members participating.

Although the main themes developed from our interviews have distinct boundaries, they mutually influence one another (indicated by the dotted lines in Fig. 1). Prioritising participation is necessary to provide continuity. Getting to know the other, feeling safe and committed to the group increase motivation to continue to participate, and all are central to finding the flow in the group. Successful communication and a sense of working together as a group are in turn key to having a positive group work experience that could make the students more inclined to prioritise further participation.

### Methodological considerations

This study is to the best of our knowledge the first to use individual interviews to gain insights into health science master’s students’ experiences with facilitators and barriers to online group work. While the study is based on a limited number of students, their various experiences with online group work, spanning from in-lecture breakout rooms to course-long work assignments, provided rich data. Choosing not to differentiate our findings according to group work format is however also a limitation as facilitators and barriers specific to format are not identified and thus implications for practice become less nuanced. Although most Norwegian students in health, social and sport sciences [51] and most European students in Health and Welfare are women [30], we consider it a limitation that only one man was recruited. As research suggests differences in collaborative styles and preferences between male and female students [52], male students may experience online group work differently than female students. Due to the gender imbalance in our recruitment, our findings are likely to better represent that latter. Another limitation is that all students were recruited from the same university in Norway. Because the students varied in age and health professional backgrounds, representing eight different health science master’s specialisations and were enrolled in an all-online course, there might still be some variation in their study contexts. However, recruitment from a single institution may have caused similarities in students experiences mainly due to similar contexts of learning created by the university’s practice and culture. Rewarding the students with a universal gift card for their participation may have introduced selection bias. Still, when asked about their reasons for participating, the students answered that they appreciated being asked to share their experiences and feeling that their opinion mattered, wanting to contribute to research in general, or being curious about what participating in an interview would involve.

Choosing a qualitative method for our study, we did not seek to generalise our results, but to gain in-depth knowledge of the master’s students’ perspectives, and as such we believe our findings offer a valuable insight into online group work in master’s programs within health science education. However, to add to our findings, online group work in health sciences should be further explored in larger and more diverse student populations, using different research designs and methods. It would also be valuable to study the different formats of online group work separately to gain a more nuanced picture. Future studies could also explore how assignments designed to promote joint work efforts influence group dynamics and learning outcomes.

### Conclusion and implications for practice.

While we acknowledge the limitations of our study, as discussed, we believe that our findings may offer guidance in the planning and design of online group work assignments in health science master’s programs. Online group work gives health science master’s students an opportunity to learn from each other’s perspectives, enhance their team-working abilities, and form a valuable community with other students. However, these benefits will be lost to the students if they do not participate or drop out due to bad group work experiences. Listening to the students in our study, we believe that online group work needs to be adapted to the specific demands of the online setting, and based on the facilitators and barriers discussed, we have several proposals. In addition to fixed groups of 3–4 students, it may be considered making elements of group assignments mandatory to ensure enough stability and continuity in the group to establish communication and efficient work processes. To promote a joint work process, rather than dividing of tasks, assignments could be designed to require the group to work together [14], and the benefits of doing so should be made clear to the students. Furthermore, to facilitate the development of good group dynamics, it may be useful to allocate time for the group to get to know each other and to introduce basic guidelines on how to work in online groups. This could include suggestions for practices to agree upon in the group, such as use of camera, choice of communication channels and digital tools and platforms, and how to organise the work processes. The aim would not be to impose a set of rules on the students, but rather to facilitate a discussion that may help them develop a work process and group dynamic that promotes learning and positive group work experiences. These efforts may also help the students understand the relevance of online group work and how they can benefit, hopefully leading to the students prioritising participation.

## Supplementary Information

Supplementary Material 1.

## Data Availability

The datasets are available from the corresponding author on reasonable request.

## Abbreviations

COREQ: Consolidated Criteria for Reporting Qualitative Studies
RTA: Reflexive Thematic Analysis
SIKT: Norwegian Agency for Shared Services in Education and Research
