# Supplementary material for: Experiences with online group work among master’s students in health sciences: a qualitative study
Source: BMC Med Educ. 2025 Sep 25;25:1262. doi: 10.1186/s12909-025-07845-w (PMC12466073; doi:10.1186/s12909-025-07845-w)
Supplement: Supplementary file 1 — Supplementary Material 1. [file 12909_2025_7845_MOESM1_ESM.pdf]

Appendix 1

Interview guide (English translation)

For qualitative individual interviews with students about their experiences with online group work and the factors that facilitate or hinder it.

1. Introductory Questions

What is the first word or phrase that comes to mind when I say "online group work"?

What do you understand by the term "online group work"?

2. Experiences with Group Work

Can you describe your experience with online group work during your master's programme so far?

What do you think contributes to successful online group work?

Can you share something you found challenging about online group work?

3. Closing Questions

Why did you decide to participate in this interview?

After a brief summary of the conversation: Does this summary seem accurate to you? Is there anything you would change? Have I understood you correctly?

Do you have any additional comments or thoughts you'd like to share?

Post-Recording Questions:

What are you currently studying?

What bachelor's degree is your current study based on?

Do you have work experience in a healthcare profession? If so, which one?

What is your age and gender?

Intervjuguide (på norsk)

For kvalitative individuelle intervjuer med studenter om hvordan de opplever digitalt gruppearbeid og hvilke faktorer som fremmer og hemmer det digitale gruppearbeidet

1. Innledende spørsmål

Hva er det første ordet/setningen du tenker på når jeg sier digitalt gruppearbeid?

Hva legger du i begrepet "digitalt gruppearbeid"?

2. Erfaringer med gruppearbeidet

Kan du fortelle litt om hvordan du har opplevd digitalt gruppearbeid på masteren så langt?

Hva opplever du bidrar til et godt digitalt gruppearbeid?

Kan du fortelle om noe du har opplevd som vanskelig i det digitale gruppearbeidet?

3. Avsluttende

Hvorfor valgte du å delta på dette intervjuet?

Etter kort oppsummering av samtalen: Synes du denne oppsummeringen passer? Er det noe du ville endret på i oppsummeringen? Har jeg forstått deg riktig?

Har du noen tilleggskommentarer eller tanker du vil supplerer med til slutt?

Etter avsluttet lydopptak:

Hva studerer du nå? Hvilken bachelor bygger nåværende studie på?

Har du arbeidserfaring fra en helseprofesjon, i så fall hvilken?

Hva er din alder og kjønn?
